# Placebo Response in Phase II-III Symptom Intervention Studies: A Focus on Chemotherapy-Induced Peripheral Neuropathy and Associated Neuropathic Pain

**DOI:** 10.3390/cancers18101514

**Published:** 2026-05-08

**Authors:** David Zahrieh, Daniel Satele, Hiboombe Haamankuli, Xin Shelley Wang, Jennifer G. Le-Rademacher, Minji Lee, Heshan Liu, Julian Diaz-Cobo, Shu-En Shen, Selina Chow, Maryam Lustberg, Kathryn J. Ruddy, Ellen M. Lavoie Smith

**Affiliations:** 1 Alliance Statistics and Data Management Center, Mayo Clinic, Rochester, MN 55905, USA; 2 Department of Quantitative Health Sciences, Mayo Clinic, Rochester, MN 55905, USA; 3 Department of Acute, Chronic, and Continuing Care, University of Alabama at Birmingham School of Nursing, Birmingham, AL 35294, USA; 4 Department of Symptom Research, The University of Texas MD Anderson Cancer Center, Houston, TX 77030, USA; 5 Alliance Protocol Operations Office, University of Chicago, Chicago, IL 60606, USA; 6 Yale Cancer Center, New Haven, CT 06520, USA; 7 Mayo Clinic, Rochester, MN 55905, USA

**Keywords:** chemotherapy-induced peripheral neuropathy, meta-analysis, placebo response, placebo-controlled double-blind clinical trial, symptom intervention

## Abstract

**Background/Objectives.** Decades of research have failed to uncover effective approaches to prevent chemotherapy-induced peripheral neuropathy (CIPN), a common side effect of neurotoxic chemotherapy. Increased interest in placebo response as a potentially under-recognized confounder in CIPN trials was recently sparked by the results of a multisite NCI-funded phase II-III CIPN prevention study of duloxetine, a promising serotonin–norepinephrine reuptake inhibitor that enhances pain-inhibitory mechanisms within the central nervous system. Study findings revealed high and nearly equivalent response rates in three randomized treatment groups—little to no CIPN was reported by 65.2%, 66.0%, and 68.0% of study participants who received duloxetine 30 mg, 60 mg, or placebo treatment, respectively. **Methods.** We performed a meta-analysis of placebo response rates from seven randomized, double-blinded, placebo-controlled trials conducted over the past 20 years and comprising 191 placebo participants that were specifically testing interventions for oxaliplatin- and paclitaxel-induced peripheral neuropathy and that serially collected patient responses on the European Organization for Research and Treatment of Cancer Quality of Life Questionnaire for Chemotherapy-Induced Neuropathy questionnaire. Additionally, we sought to identify trial- and patient-specific factors that predicted higher placebo response rates from a participant-level pooled analysis. **Results.** The placebo response rate was 10.0% [95% CI: 5.8%, 16.6%] when response was defined more conservatively as patients reporting no neuropathy at all. When the placebo response was defined more broadly based on patients reporting no or a little neuropathy, the placebo response rate was higher (39.6% [95% CI: 27.4%, 53.2%]). Male participants, receipt of oxaliplatin, and a 2:1 randomization ratio favoring the intervention arm were individually associated with a higher placebo response. **Conclusions.** High placebo response rates can threaten scientific progress toward identifying effective treatments for cancer treatment-associated side effects, like CIPN. Careful attention to study design factors, participant eligibility, and patient and research staff expectations may help to minimize placebo response rates in future CIPN intervention studies.

## Simple Summary

Numerous scientific challenges in prior chemotherapy-induced peripheral neuropathy (CIPN) studies compromised our success in finding effective strategies to prevent CIPN, including potentially high placebo response rates. Findings from a multisite National Cancer Institute (NCI)-funded phase II-III CIPN prevention study of duloxetine, a promising serotonin–norepinephrine reuptake inhibitor, revealed high and nearly equivalent response rates in the three randomized treatment groups. Little to no CIPN was reported by 65.2%, 66.0%, and 68.0% of participants who received duloxetine 30 mg, 60 mg, or placebo treatment, respectively. While duloxetine was not shown to be more effective than placebo in preventing CIPN, perhaps duloxetine’s efficacy could not be proven due to factors that artificially enhanced placebo response. Taken together, placebo response might have been a factor in other CIPN trials, possibly explaining, in part, why CIPN prevention trials have failed. Given these provocative results, we report the findings of a meta-analysis from seven phase II/III CIPN randomized, placebo-controlled prevention studies. The findings can inform future placebo response mitigation strategies to optimize clinical trial success.

## 1. Introduction

Chemotherapy-induced peripheral neuropathy (CIPN) is a frequently encountered side effect of neurotoxic chemotherapy [1,2]. Characterized by numbness, tingling, and neuropathic pain, CIPN affects up to 80% of patients receiving taxanes, platinum analogs, vincas, and other chemotherapy agents [3,4,5]. Despite years of intervention research, there is currently no effective intervention that can prevent CIPN [6]. Six Factors, such as limited knowledge of CIPN mechanisms, minimal attention to genetic predisposition, lack of gold-standard CIPN measurement approaches, poor control for confounding variables, insensitive primary endpoints, and threats to statistical validity, have compromised efforts to discover effective treatments [7].

Compounding the lack of scientific advancement regarding CIPN prevention is the high rate of placebo-effect responses in clinical trials. A placebo effect occurs whenever there is a physiological or psychological improvement from receiving a matching placebo that is identical in appearance to the intervention [8,9]. The current literature suggests that over 50% of treatment effects in clinical trials are often attributed to the placebo [10,11]. When placebo response rates are high, it is challenging to show that promising new treatments are better than no treatment at all, even when the intervention is effective. The following predictors of placebo response have been identified: expectations and learning mechanisms; biological and genetic factors; psychological and contextual influences; clinical trial design features; participant characteristics; cultural and social factors; and participants’ clinical conditions [12,13,14,15]. Together, these factors reflect the complex interplay of psychological, biological, social, and methodological influences that should be considered when designing placebo-controlled CIPN prevention trials. However, we found no published studies providing insight or strategies for mitigating the placebo response in placebo-controlled CIPN intervention studies.

Recent interest in placebo responses in CIPN trials emerged from a National Cancer Institute (NCI)-funded phase II-III study of duloxetine, a promising serotonin–norepinephrine reuptake inhibitor, which demonstrated high response rates across all treatment groups, including the placebo group where patients received a matching placebo for duloxetine that was identical in appearance; in this setting of symptom prevention, by response we mean successful prevention of CIPN. In this study, little to no CIPN was reported by 65.2%, 66.0%, and 68.0% of participants receiving duloxetine 30 mg, 60 mg, or placebo, respectively [16]. Given these results, our objective was to perform a meta-analysis of data from phase II and III CIPN randomized, double-blinded, placebo-controlled intervention studies conducted over the past 20 years from the NCI-National Clinical Trial Network (NCTN) that incorporated the European Organization for Research and Treatment of Cancer (EORTC) Quality of Life Questionnaire for Chemotherapy-Induced Neuropathy (QLQ-CIPN20) to quantify the placebo response rate. Additionally, we sought to identify trial- and patient-specific factors that predicted higher placebo response rates from a participant-level pooled analysis. The findings will inform future strategies to mitigate placebo response and optimize clinical trial success.

## 2. Methods

### 2.1. Trial and Participant Selection

From the 28 primary trials for prevention of CIPN that were presented in the 2020 American Society of Clinical Oncology (ASCO) guideline update [6], plus Alliance for Clinical Trials in Oncology (Alliance) A221805, seven randomized, double-blinded, placebo-controlled trials specifically testing interventions for oxaliplatin- and paclitaxel-induced peripheral neuropathy and that serially collected patient responses on the EORTC QLQ-CIPN20 questionnaire were identified. Patient-level data from patients who were randomized to the placebo arms of these seven trials were included. A synopsis of each trial is provided (Table 1A), and the primary results from each trial were published in the past 12 years (between 2014 and 2026) [16,17,18,19,20,21,22]. Three of the seven trials were conducted through the NCI-NCTN (North Central Cancer Treatment Group (NCCTG) N08CA and N08CB, and Alliance A221805). NCCTG is now part of the Alliance. Of the remaining four trials, two were industry-sponsored through the Academic and Community Cancer Research United (ACCRU) collaborative network (RC11C3 and RU221408I), one was conducted through the Mayo Clinic Comprehensive Cancer Center (MCCCC) (MC11C4), and one was conducted at the MD Anderson Cancer Center (MD Anderson). The MCCCC Institutional Review Board (IRB) reviewed this study and determined that the planned analysis was exempt from IRB approval due to the use of de-identified data. The staff at the Statistics and Data Management Center responsible for each respective trial prepared individual-level de-identified data that were formatted to comply with HIPAA patient confidentiality stipulations and follow the best practices described in the NCTN Program Data Sharing Policy. The de-identified data sets were then shared with the study team.

### 2.2. Co-Primary Response Endpoints

**Figure 1:**
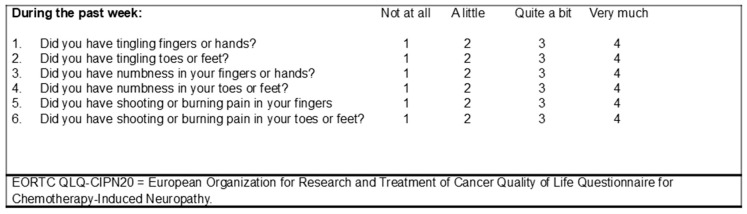
Sensory symptoms corresponding to the first six items on the EORTC QLQ-CIPN20 questionnaire.

Two co-primary response endpoints were considered: (1) a patient reporting that sensory symptoms had affected them “Not at all”; (2) a patient reporting that sensory symptoms had affected them “Not at all” or “A little”. Both co-primary endpoints represented a composite response reflecting sensory CIPN symptom severity and onset measured on Day 1 of each chemotherapy cycle during the first 3 months of treatment, as well as 1–3 weeks after the 3-month dose, using six items from the EORTC QLQ-CIPN20 [22,23,24,25] that quantify numbness (N), tingling (T), and pain in the fingers (or hands) and toes (or feet). For each of the six individual questions, patients were asked to select 1 of 4 choices regarding how each of the sensory symptoms had affected them during the preceding week: 1 = Not at all, 2 = A little, 3 = Quite a bit, and 4 = Very much (Figure 1). Because we expected that CIPN onset would be variable and assumed that each sensory symptom is similarly important clinically, we calculated the highest (worst) N, T, and pain sensory score obtained anytime during oxaliplatin or paclitaxel exposure over a 3-month time period, including 1–3 weeks after the 3-month dose. The first co-primary response endpoint was defined as a patient reporting a highest score of 1 (“Not at all”). The second co-primary response endpoint was defined as a patient reporting a highest score of 1 (“Not at all”) or 2 (“A little”). The latter co-primary response endpoint was the primary response endpoint used in the design and analysis of the motivating phase II-III study of duloxetine (Alliance A221805) [16].

### 2.3. Analysis Population

For the two co-primary response endpoints, the analyses were performed on a modified full analysis set, defined as all randomized and eligible patients who received 3 months of oxaliplatin or paclitaxel and completed the six QLQ-CIPN20 sensory symptom items (i.e., N, T, and pain in the fingers/hands and toes/feet). The analyzable patients contributed QLQ-CIPN20 data on at least 3 post-baseline (Day 1 Cycle 1) occasions, with one assessment occurring 1–3 weeks after completion of the 3-month time point. The motivation for defining the analysis population in this way was twofold. First, it ensured that the analyzable patients were exposed to a cumulative dosage of neurotoxic chemotherapy sufficient to cause CIPN when measured using a patient-reported outcome measure (Table 1B). Moreover, 3 months of oxaliplatin or paclitaxel is the minimum recommended treatment duration to reduce the risk of disease relapse in this patient population [22,23,24,25,26]. Second, it ensured that the analyzable patients were able to provide patient-reported feedback during cumulative exposure (i.e., on ≥2 occasions) and during the early post-treatment period (1–3 weeks after receiving 3 months of neurotoxic drug treatment).

### 2.4. Statistical Analysis

The distribution of participant baseline and trial design factors was summarized and compared between the participants who comprised the analysis population versus (1) the participants who were excluded and (2) the participants who received 3 months of neurotoxicity chemotherapy but who were excluded specifically because they had insufficient survey data using Fisher’s exact test for categorical variables and the Wilcoxon rank-sum test for continuous variables. Additionally, the percentage of patients in the analysis population who continued to receive a full dose of oxaliplatin or paclitaxel by 3 months was reported.

To bring together the placebo response rates from the placebo arms of the seven trials, while accounting for trial-to-trial variation, a formal meta-analysis was performed [27]. A generalized linear mixed model with logit transformation was used to estimate the overall placebo response and 95% confidence interval (CI) for each co-primary endpoint. The SAS NLMIXED procedure was used with Gauss–Hermite quadrature to approximate the likelihood function in obtaining maximum likelihood estimates. The number of quadrature points used was 200 and was chosen based on monitoring the convergence of estimates and standard errors.

The data were then pooled across the seven trials, and univariate associations with placebo response were assessed for each participant’s baseline and trial design factor using Fisher’s exact test for categorical variables and the Wilcoxon rank-sum test for continuous variables. The participant factors considered were uniformly collected on the case report forms for each of the seven trials and included age (in years), sex assigned at birth, ethnicity, self-reported race, Eastern Cooperative Oncology Group (ECOG) performance status (PS), cancer site, neurotoxic agent received, years between enrollment and 1 January 2026, and insurance payment method. Additionally, we identified the county where each participant resided and used the Economic Research Service to ascertain the county-level socioeconomic covariates, percent in poverty in 2023 (0–100%), and the unemployment rate in 2013 (0–100%), as well as the rural–urban continuum code from 2013 (range: 1–9 corresponding to large metro areas to completely rural). Trial factors included the length of trial (defined as the date the last participant enrolled minus the date the first participant enrolled divided by 365.25), the randomization ratio used to randomly allocate participants to the experimental therapy or placebo, and the size of the trial (≥60 placebo patients versus <60).

Analyses were performed in SAS v9.4 at the Alliance Statistics and Data Management Center. *p* values were two-sided and reported as continuous quantities. No adjustment for multiple testing was made. A nominal *p* < 0.050 was considered statistically significant.

## 3. Results

### 3.1. Baseline Characteristics

**Figure 2:**
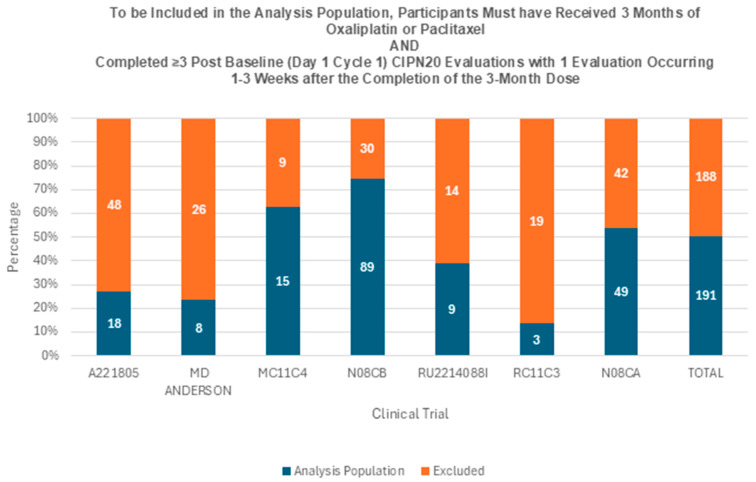
Number of placebo patients for each of the seven trials and overall who met the criteria to be included in the analysis population. QLQ-CIPN20 = European Organization for Research and Treatment of Cancer (EORTC) Quality of Life Questionnaire (QLQ) for Chemotherapy-Induced Neuropathy (CIPN).

Of the 379 placebo patients who received ≥1 dose of oxaliplatin or paclitaxel in any of the seven trials, 191 (50%) received 3 months of oxaliplatin or paclitaxel and completed the six QLQ-CIPN20 sensory symptom items (i.e., N, T, and pain in the fingers/hands and toes/feet) on three or more occasions after baseline (Day 1 Cycle 1), with one assessment occurring 1–3 weeks after the completion of the 3-month dose, which comprised the analysis population (Figure 2). Of these, 172 (90.1%) were White; 14 (7.3%) were Black or African American; and five (2.6%) were of another race. The median age was 58 years old; 65% were female; and 38.2% had an ECOG PS of 1 or 2, while 57.6% had an ECOG PS of 0 (Table 2).

Patient characteristics were largely similar between patients included and excluded from the analysis population (Table 2). Compared with the 79 patients who received 3 months of neurotoxic chemotherapy but had incomplete survey data, the analyzable patients had a lower proportion of Hispanics or Latinos (4.2% vs. 11.4%) and a higher proportion of gastrointestinal cancers (66.5% vs. 50.6%) coupled with a lower proportion of cancers other than breast, cervical, ovarian, and uterine (7.9% vs. 20.3%); additionally, the analyzable patients enrolled earlier (median of 15 years ago vs. 12 years ago). Similar differences were seen when comparing the patients included in the analysis population to all 188 patients excluded because they did not meet either inclusion criterion (Table 2).

### 3.2. Use of Neurotoxic Agent

In the 158 patients with dosing information available (128 oxaliplatin; 30 paclitaxel), the proportion of patients still receiving a full dose of the neurotoxic agent (oxaliplatin or paclitaxel) at 3 months was 77.2%. The percentage receiving full dose oxaliplatin at 3 months was 76.6%, which was similar to the 80.0% still receiving full dose paclitaxel at 3 months.

### 3.3. Meta-Analysis

For the analyzable patients, Table 3A,B show the trial-by-trial observed placebo response rates for each co-primary response endpoint as well as the corresponding overall meta-analytic response rate with a 95% CI. The overall meta-analytic estimate was 10.0% [95% CI: 5.8%, 16.6%] for a placebo response defined as a highest CIPN sensory symptom score of 1 (Not at all). For a placebo response defined as a highest CIPN sensory symptom score of 1 (Not at all) or 2 (A little), the overall estimate was 39.6 [95% CI: 27.4%, 53.2%]. For each co-primary response endpoint, the overall estimate remained qualitatively the same when the largest trial, NCCTG N08CB (*N* = 89), was omitted (10.8% [5.1%, 21.5%] and 38.7% [22.3%, 58.2%], respectively), although the CIs were wider.

The meta-analysis was repeated for each response endpoint after excluding Alliance A221805, the trial that motivated the current research. The overall meta-analytic estimate was 8.7% [4.5%, 16.0%] for a response defined as a highest CIPN sensory symptom score of 1 (Not at all). The corresponding observed response rate in Alliance A221805 was 22.2%, which is to the right of the upper bound of the 95% CI. For the response endpoint defined as a highest CIPN sensory symptom score of 1 (Not at all) or 2 (A little), the overall meta-analytic estimate was 36.3% [24.9%, 49.4%], and again, the observed response rate in Alliance A221805 of 61.1% was larger than the upper bound of the 95% CI.

### 3.4. Univariate Associations

A pooled analysis was performed to identify individual participant- and trial-level factors that predicted placebo responses. Of the 191 analyzable patients across the seven trials, 19 (9.9%) reported a highest score of 1 (Not at all), and 75 (39.3%) reported a highest score of 1 (Not at all) or 2 (A little). As a sensitivity analysis on the pooled data, a comparison was made to the patients who (1) did not meet either or both criterion for inclusion in the analysis population but provided some QLQ-CIPN20 sensory symptom data (N = 155) and (2) received 3 months of neurotoxic chemotherapy and provided some QLQ-CIPN20 sensory symptom data, albeit an insufficient amount to be included in the analysis population (N = 73). In comparison to the patients who did not meet the criteria for inclusion in the analysis population and who also completed the six QLQ-CIPN20 sensory symptom items on at least one occasion (N = 155), the percentage reporting a highest score of 1 (Not at all) was similar (11.0% vs. 9.9%; *p* = 0.757); however, the percentage reporting a highest score of 1 (Not at all) or 2 (A little) was higher in the patients who were excluded (54.8% vs. 39.3%; *p* = 0.004). In comparison to the patients who were excluded despite receiving 3 months of neurotoxic chemotherapy but submitted some survey data (N = 73), the percentage reporting a highest score of 1 (Not at all) was also similar (13.7% vs. 9.9%; *p* = 0.383) and the percentage reporting a highest score of 1 (Not at all) or 2 (A little) was also higher in the patients who were excluded (64.4% vs. 39.3%; *p* = 0.003).

While there was a lack of evidence of an association between participant- and trial-level factors and response, defined as a highest CIPN sensory symptom score of 1 (Not at all) (Table 4A), sex assigned at birth, cancer site, neurotoxic agent, and the trial’s randomization ratio were significantly associated with placebo response defined as a highest CIPN sensory symptom score of 1 (Not at all) or 2 (A little) (Table 4B). Specifically, a higher proportion of males reported a response compared with females (53.5% vs. 32.0%; *p* = 0.005), and a lower proportion of patients with breast, cervical, ovarian, or uterine cancer reported a response (24.5%) compared to patients with gastrointestinal (44.1%) or other cancer (46.7%) (*p* = 0.048). Also, patients receiving oxaliplatin reported a higher response rate compared to patients receiving paclitaxel (46.2% vs. 24.6%; *p* = 0.004). Notably, patients on placebo who participated in a trial that randomized patients in a 2:1 ratio favoring the experimental agent reported a higher response rate compared with patients on placebo who participated in a trial that randomized patients to either the experimental agent or placebo in a 1:1 fashion (45.8% vs. 31.0%; *p* = 0.037).

## 4. Discussion

High placebo response rates (>50%) are commonly reported in symptom intervention clinical trials. This meta-analysis of seven CIPN intervention studies revealed a 10.0% [95% CI: 5.8%, 16.6%] to 39.6% [95% CI: 27.4%, 53.2%] placebo response rate, depending on how strictly we defined “response”. There was a lower (10.0%) placebo response rate when response was defined more conservatively as patients reporting no neuropathy at all. When the placebo response was defined more broadly based on patients reporting no or a little neuropathy, the placebo response rate was higher (39.6%). Regardless of how placebo response was defined, the overall rates identified in this meta-analysis were not unusually high (>50%).

It is important to note that the observed placebo response in one trial (22.2% and 61.1% in Alliance A221805) was above the corresponding upper bound of the 95% CI calculated based on the other six trials— [95% CI: 4.5%, 16.0%] and [95% CI: 24.9%, 49.4%]—irrespective of how the placebo response was defined. This suggests that unique factors may have influenced participants’ responses in this trial. Alliance A221805 placebo response rates were potentially influenced by every predictor of high response identified in the pooled analysis; there was a higher proportion of male to female participants, all patients received oxaliplatin to treat a gastrointestinal cancer, and the trial design incorporated an overall 2:1 allocation ratio to duloxetine (30 mg or 60 mg) versus placebo. Knowledge of the unequal allocation favoring duloxetine may have created a heightened expectation that the patient was receiving duloxetine and, therefore, led to an increased placebo response [28].

Beyond the measurable placebo response predictors that were identified in the univariate analysis, we hypothesize that other unmeasurable variables may have influenced the high placebo response rate in Alliance A221805. Hope for duloxetine’s potential may have been high among both patients, study staff, and treating physicians because, despite decades of research in this area, there are still no known preventative treatments. The oncology community is desperate to find a new way to help patients avoid CIPN-associated suffering.

Another factor that is unique to the Alliance A221805 trial that could explain the higher placebo response in this trial, when compared to the other six studies included in the analysis, is that our first landmark duloxetine trial demonstrating efficacy for established painful CIPN [3] was conducted within the same NCI-supported clinical trials network (previously Cancer and Leukemia Group B [CALGB]) as Alliance A221805 (CALGB merged with two other groups to form the Alliance). Thus, Alliance members may have been eager to support this second trial due to familiarity with the study team and their prior success, and this could have fueled high hopes that this second duloxetine trial would also reveal duloxetine efficacy to prevent CIPN. This optimism could have been unintentionally conveyed to study participants to a greater degree than what we might see in the other studies, potentially influencing placebo responses, because two studies of duloxetine were conducted within the same research network, where members were aware of the previous positive duloxetine findings and familiar with and supportive of the study team.

Another unique, unmeasurable influencing variable may have been linked to the study staff support of Alliance A221805 by a dedicated nurse liaison. In 2025, we published the results of a qualitative study exploring the communication, education, and support strategies used by a dedicated nurse liaison who was uniquely assigned to support Alliance A221805 [29]. The nurse liaison was an experienced nurse practitioner who had the sole responsibility of supporting all Alliance A221805 participating sites, mainly through remote contact. We conducted a qualitative analysis of 1500 email messages sent/received by the liaison and staff from 73 participating sites over the study duration (1 May 2020 to 21 March 2023). To our knowledge, this level of dedicated support was not implemented in any of the other six studies included in this meta-analysis. The qualitative findings suggested that the nurse liaison’s communication was educational, validating of study staff knowledge and approaches, and caring (defined as accessible, contributive, and considerate). Over time, the nurse liaison developed strong collaborative relationships with site staff and physicians, and anecdotally, optimism for the trial was common. Thus, it is possible that nurse liaison support, while potentially critical for enhancing study staff satisfaction, knowledge, and confidence, patient safety, and research fidelity may have contributed to a higher placebo response rate in Alliance A221805 when compared to the other six studies.

The rigorous meta-analysis has several strengths. Meta-analyses provide the highest level of scientific evidence by virtue of the inherently rigorous analytic methods [30]. The findings are highly generalizable because data were obtained from a geographically diverse sample of patients who were enrolled in neuropathy trials nationwide. Data were carefully matched across the seven studies to achieve a homogeneous sample of patients who received either oxaliplatin or paclitaxel over the same treatment time (3 months), and CIPN outcomes were assessed using the same validated primary outcome measure (QLQ-CIPN20) and number of assessments that spanned the study duration (baseline to 1–3 weeks after the last 3-month chemotherapy treatment). Controlling for chemotherapy treatment duration was a particularly important approach that allowed further consideration of a main criticism of Alliance A221805; patients received a lower oxaliplatin cumulative dose (3 months vs. 6 months of treatment) and, consequently, would not develop severe CIPN. To address this concern, we controlled for cumulative oxaliplatin dosage. More specifically, in the four included studies of oxaliplatin-induced CIPN (including Alliance A221805), we only included CIPN responses obtained at the 3-month time point when all patients had received the same cumulative dosage of 510–520 mg/m^2^.

In any event, two sensitivity analyses were performed on the pooled data. One potential concern is that patients who received 3 months of neurotoxic chemotherapy but who were excluded from the primary analysis population because they submitted an insufficient amount of survey data may have failed to complete surveys because they experienced worse CIPN. However, the observed data do not support this explanation. A higher proportion of these patients excluded reported minimal symptoms, defined as a highest score of 1 (Not at all) or 2 (A little), compared with patients in the analysis population (64.4% vs. 39.3%, respectively), suggesting that not completing the surveys was not driven by worse CIPN. Another potential concern is that patients excluded from the analysis population may have had insufficient chemotherapy exposure and/or failed to complete surveys because they experienced worse CIPN. Among all the placebo-treated patients excluded from the analysis population, worse neuropathy symptoms were not reported. On the contrary, a significantly higher proportion of the excluded patients reported minimal symptoms, defined as a highest score of 1 (not at all) or 2 (A little), compared with patients included in the analysis population (54.8% vs. 39.3%, respectively), suggesting that exclusion was not driven by worse CIPN.

Several limitations potentially compromise the internal validity of this study. Demographic variables were compared between included and excluded cases, revealing differences between the two groups that should be considered. When compared to excluded cases, there were fewer Hispanic patients and more patients with gastrointestinal cancer included in the analysis population. Also, there were more patients in the analysis group who were enrolled in more recently conducted studies and on trials using an overall 2:1 allocation ratio. These differences compromise the ability to generalize the meta-analysis findings to Hispanic populations and to patients with other cancer types. Further, since trial duration and randomization ratio are factors that can influence placebo response rates, the univariate analysis may have overestimated the strength of the associations among these variables. Additionally, unpublished trials or trials published after the 2020 ASCO guideline were not considered in the current research. Furthermore, the neurotoxic agents included in the study were limited to oxaliplatin and paclitaxel, and the results may not extend to CIPN caused by other neurotoxic agents.

## 5. Conclusions

In conclusion, high placebo response rates can threaten scientific progress toward identifying effective treatments for cancer treatment-associated side effects, like CIPN. Careful attention to study design factors such as use of objective outcome measures, statistical methods, participant eligibility, and patient and research staff expectations may help to minimize placebo response rates in future CIPN intervention studies.

## Figures and Tables

**Table 1:** (**A**) Synopsis of the seven randomized, double-blinded, placebo-controlled trials [16,17,18,19,20,21,22] that incorporated serial assessments from the patient-reported EORTC QLQ-CIPN20 questionnaire. (**B**) Cycles and cumulative doses of oxaliplatin and paclitaxel over 3 months.

(A)						
Neurotoxic Agent	Trial	Year Published	Experimental Agent	Randomization Ratio [Exp: Placebo]	Trial Phase	Number of Patients Exposed to Placebo
**Oxaliplatin**	A221805 [13]	2026	Duloxetine	2:1	II–III	66
	MD Anderson [15]	2019	Minocycline	1:1	II	34
	MC11C4 [16]	2016	Venlafaxine	1:1	II	24
	N08CB [17]	2014	Ca/Mg	2:1	III	119
**Paclitaxel**	RU2214088I [18]	2017	Minocycline	1:1	II	23
	RC11C3 [19]	2016	Pregabalin	1:1	II	22
	N08CA [20]	2014	Glutathione	1:1	III	91
**TOTAL**	**7**	**TOTAL**				**379**
(**B**)						
**OXALIPLATIN ** **FOLFOX**			**OXALIPLATIN ** **CAPOX**			
**Cycle**	**Cumulative Weeks**	**Cumulative** **Dose**	**Cycle**	**Cumulative Weeks**	**Cumulative** **Dose**	
Cycle 1	2	85 mg/m^2^	Cycle 1	3	130 mg/m^2^	
Cycle 2	4	170 mg/m^2^	Cycle 2	6	260 mg/m^2^	
Cycle 3	6	255 mg/m^2^	Cycle 3	9	390 mg/m^2^	
Cycle 4	8	340 mg/m^2^	Cycle 4	12 (3 Months)	520 mg/m^2^	
Cycle 5	10	425 mg/m^2^	Day 1 Cycle 5	FINAL CIPN20		
Cycle 6	12 (3 Months)	510 mg/m^2^				
Day 1 Cycle 7	FINAL CIPN20					
**PACLITAXEL**						
**Cycle**	**Cumulative Weeks**	**Cumulative** **Dose**	**Cycle**	**Cumulative Weeks**	**Cumulative Dose**	
Cycle 1	1	80 mg/m^2^	Cycle 7	7	560 mg/m^2^	
Cycle 2	2	160 mg/m^2^	Cycle 8	8	640 mg/m^2^	
Cycle 3	3	240 mg/m^2^	Cycle 9	9	720 mg/m^2^	
Cycle 4	4	320 mg/m^2^	Cycle 10	10	800 mg/m^2^	
Cycle 5	5	400 mg/m^2^	Cycle 11	11	880 mg/m^2^	
Cycle 6	6	480 mg/m^2^	Cycle 12	12 (3 Months)	960 mg/m^2^	
			Day 1 Cycle 13	FINAL CIPN20		

EORTC QLQ-CIPN20 = European Organization for Research and Treatment of Cancer Quality of Life Questionnaire for Chemotherapy-Induced Neuropathy. Ca = calcium; Mg = magnesium. Note: The ClinicalTrials.gov ID for Alliance A221805, MD Anderson, MC11C4, N08CB, RU2214088I, RC11C3, and N08CA are NCT04137107, NCT01906008, NCT01611155, NCT01099449, NCT02297412, NCT01637077, and NCT02311907, respectively.

**Table 2:** Baseline characteristics for (1) the analysis population, (2) those excluded from the analysis population specifically because they completed insufficient survey data despite having received 3 months of neurotoxic chemotherapy, and (3) all participants who were excluded because they did not meet either or both criteria.

	Analysis Population * N = 191	Comparison with the Analysis Population			
		3 Months of Neurotoxic Chemotherapy but Insufficient Survey Data		Did Not Meet Either or Both Criteria for Inclusion	
Baseline Factor		N = 79	*p*	N = 188	*p*
**Age, years**	191	79	0.888	188	0.332
N	58.0	56.0		58.0	
Median (Q1, Q3)	(48.0, 64.0)	(50.0, 64.0)		(50.0, 67.0)	
**Female, n (%)**	125 (65.4%)	50 (63.3%)	0.736	113 (60.1%)	0.282
**Hispanic or Latino, n (%)**	8 (4.2%)	9 (11.4%)	0.046	19 (10.1%)	0.031
**ECOG PS, n (%)**					
0	110 (57.6%)	47 (59.5%)	0.276	110 (58.5%)	0.287
1	69 (36.1%)	18 (22.8%)		48 (25.5%)	
2	4 (2.1%)	2 (2.5%)		4 (2.1%)	
Unknown	8 (4.2%)	12 (15.2%)		26 (13.8%)	
**Race, n (%)**					
White	172 (90.1%)	64 (81.0%)	0.820	165 (87.8%)	0.283
Black or African American	14 (7.3%)	9 (11.4%)		12 (6.4%)	
Other	5 (2.6%)	6 (7.6%)		11 (5.9%)	
**Cancer site, n (%)**					
Breast/cervical/ovarian/uterine	49 (25.7%)	23 (29.1%)	0.007	53 (28.2%)	<0.001
GI	127 (66.5%)	40 (50.6%)		96 (51.1%)	
Other	15 (7.9%)	16 (20.3%)		39 (20.7%)	
**Neurotoxic agent, n (%)**					
Paclitaxel	61 (31.9%)	30 (38.0%)	0.340	75 (39.9%)	0.106
Oxaliplatin	130 (68.1%)	49 (62.0%)		113 (60.1%)	
**Years since enrollment (from January 2026)**					
N	191	79	<0.001	188	<0.001
Median (Q1, Q3)	15.0 (14.0, 15.0)	12.0 (5.0, 15.0)		13.0 (6.0, 15.0)	
**Insurance payment method, n (%)**					
Medicaid or Medicare/Medicaid	7 (3.7%)	6 (7.6%)	0.083	13 (6.9%)	0.067
Medicare or Medicare/private insurance	37 (19.4%)	17 (21.5%)		47 (25.0%)	
Military-sponsored (including CHAMPUS and TRCARE)	0 (0.0%)	2 (2.5%)		3 (1.6%)	
No means of payment (no insurance)	5 (2.6%)	0 (0.0%)		3 (1.6%)	
Other	1 (0.5%)	0 (0.0%)		0 (0.0%)	
Private insurance	123 (64.4%)	40 (50.6%)		90 (47.9%)	
Self-pay (no insurance)	7 (3.7%)	2 (2.5%)		6 (3.2%)	
Unknown	11 (5.8%)	12 (15.2%)		26 (13.8%)	
**Length of trial, years [last enrolled—first]**					
N	191	79	<0.001	188	<0.001
Median (Q1, Q3)	1.7 (1.7, 1.8)	1.8 (1.7, 2.8)		1.8 (1.7, 2.8)	
**County-level characteristics, N, median (Q1, Q3)**					
Percent in poverty 2023 (0–100%)	182 11.7 (9.2, 14.1)	67 11.9 (9.9, 15.2)	0.593	159 11.9 (9.8, 15.2)	0.461
Unemployment rate in 2013 (0–100%)	182 6.9 (5.5, 8.4)	67 7.0 (5.0, 8.3)	0.607	161 7.0 (5.6, 8.4)	0.801
Rural-Urban Continuum Code 2013 (1, 2, …, 9)	182 2.0 (1.0, 3.0)	67 2.0 (1.0, 4.0)	0.963	159 2.0 (1.0, 3.0)	0.483
**Randomization ratio, n (%)**					
2:1 (favoring experimental arm)	107 (56.0%)	35 (44.3%)	0.079	78 (41.5%)	0.005
1:1	84 (44.0%)	44 (55.7%)		110 (58.5%)	
**Size of trial**					
≥60 patients (N08CA, N08CB, A221805)	156 (81.7%)	53 (67.1%)	0.009	120 (63.8%)	<0.001
<60 patients	35 (18.3%)	26 (32.9%)		68 (36.2%)	

Note: *p* values are two-sided and based on Fisher’s exact test for categorical variables and the Wilcoxon rank-sum test for continuous features. Not reported or unknown were excluded from statistical tests. * To be included in the analysis population, participants needed to have received 3 months of oxaliplatin or paclitaxel and completed the six QLQ-CIPN20 sensory symptom items (i.e., N, T, and pain in the fingers/hands and toes/feet) on at least three post-baseline (Day 1 Cycle 1) occasions, with one assessment occurring 1–3 weeks after completion of the 3-month time point.

**Table 3:** (**A**) Observed placebo responses (highest CIPN sensory symptom score 1 = Not at all) by trial and the overall meta-analytic estimate with the 95% confidence interval. (**B**) Obsered lpacebo responses (highest CIPN sensory symptom score 1 = Not at all or 2 = A little) by trial and the overall meta-analytic estimate with the 95% confidence interval.

**(A)**					
**Neurotoxic** **Agent**	**Publication Year**	**Trial**	**Observed No. of Placebo Responses**	**No. of Patients**	**Percentage of Placebo Responders**
Oxaliplatin	2026	A221805 [13]	4	18	22.22%
	2019	MD Anderson [15]	2	8	25.00%
	2016	MC11C4 [16]	1	15	6.67%
	2014	N08CB [17]	8	89	9.00%
Paclitaxel	2017	RU221408I [18]	0	9	0.00%
	2016	RC11C3 [19]	0	3	0.00%
	2014	N08CA [20]	4	49	8.16%
**Overall meta-analytic estimate [95% CI]**					**9.95% [5.76%, 16.60%]**
(**B**)					
**Neurotoxic** **Agent**	**Publication Year**	**Trial**	**Observed No. of Placebo Responses**	**No. of Patients**	**Percentage of Placebo Responders**
Oxaliplatin	2026	A221805 [13]	11	18	61.11%
	2019	MD Anderson [15]	4	8	50.00%
	2016	MC11C4 [16]	7	15	46.67%
	2014	N08CB [17]	38	89	42.70%
Paclitaxel	2017	RU221408I [18]	2	9	22.22%
	2016	RC11C3 [19]	1	3	33.33%
	2014	N08CA [20]	12	49	24.49%
**Overall meta-analytic estimate [95% CI]**					**39.60% [27.42%, 53.23%]**

**Table 4:** (**A**) Univariate associations between participant- or trial-level factor and placebo response defined as a highest CIPN sensory symptom score of 1 (Not at all). (**B**) Univariate associations between participant- or trial-level factor and placebo response defined as a highest CIPN sensory symptom score of 1 (Not at all) or 2 (A little).

**(A)**				
**Baseline** **Factor**	**Placebo Response** **Highest Score** **Not at All** **N = 19**	**No Placebo Response** **Highest Score** **2, 3, or 4** **N = 172**	**Total**	***p***
**Age, years** N Median (Q1, Q3)	19 55.0 (44.0, 62.0)	172 58.0 (49.0, 64.0)	191	0.342
**Sex, n (%)** Female Male	11 (8.8) 8 (12.1)	114 (91.2%) 58 (87.9)	125 66	0.457
**Ethnicity, n (%)** Hispanic or Latino Not Hispanic or Latino Unknown/not reported	2 (25.0%) 16 (9.1%) 1 (12.5%)	6 (75.0%) 159 (90.9%) 7 (87.5%)	8 175 8	0.332
**Race, n (%)** White Black or African American Other	17 (9.9%) 2 (14.3%) 0 (0.0%)	155 (90.1%) 12 (85.7%) 5 (100%)	172 14 5	0.655
**ECOG PS, n (%)** 0 1 2 Unknown	11 (9.7%) 7 (9.7%) 0 (0.0%) 1 (50.0%)	102 (90.3%) 65 (90.3%) 4 (100.0%) 1 (50.0%)	113 72 4 2	0.807
**Cancer Site, n (%)** Breast/cervical/ovarian/uterine GI Other	3 (6.1%) 13 (10.2%) 3 (20.0%)	46 (93.9%) 114 (89.8%) 12 (80.0%)	49 127 15	0.286
**Neurotoxic agent, n (%)** Paclitaxel Oxaliplatin	4 (6.6%) 15 (11.5%)	57 (93.4%) 115 (88.5%)	61 130	0.284
**Years since enrollment (from January 2026)** N Median (Q1, Q3)	19 14.0 (11.0, 15.0)	172 15.0 (14.0, 15.0)	191	0.216
**Insurance payment method**, **n (%)** Medicaid or Medicare/Medicaid Medicare or Medicare/private insurance No means of payment (no insurance) Other Private insurance Self-pay (no insurance) Unknown	1 (14.3%) 2 (5.4%) 1 (20.0%) 0 (0.0%) 10 (8.1%) 2 (28.6%) 3 (27.3%)	6 (85.7%) 35 (94.6%) 4 (80.0%) 1 (100.0%) 113 (91.9%) 5 (71.4%) 8 (72.7%)	7 37 5 1 123 7 11	0.404
**Length of trial, years [last enrolled—first]** N Median (Q1, Q3)	19 1.8 (1.7, 2.7)	172 1.7 (1.7, 1.8)	191	0.061
**County-level characteristics, N, median (Q1, Q3)** Percent in poverty 2023 (0–100%) Unemployment rate in 2013 (0–100%) Rural-Urban Continuum Code 2013 (1, 2, …9)	17 11.4 (9.1, 13.2) 17 7.4 (5.3, 8.3) 17 2.0 (1.0, 2.0)	165 11.7 (9.2, 14.2) 165 6.9 (5.5, 8.4) 165 2.0 (1.0, 3.0)	182 182 182	0.642 0.798 0.288
**Randomization ratio, n (%)** 2:1 (favoring experimental arm) 1:1	12 (11.2%) 7 (8.3%)	95 (88.8%) 77 (91.7%)	107 84	0.509
**Size of trial** ≥60 patients (N08CA, N08CB, A221805) <60 patients	16 (10.3%) 3 (8.6%)	140 (89.7%) 32 (91.4%)	156 35	0.763
**(B)**				
**Baseline** **Factor**	**Placebo Response** **Highest Score** **Not at All or A Little** **N = 75**	**No Placebo Response** **Highest Score** **3 or 4** **N = 116**	**Total**	***p***
**Age, years** N Median (Q1, Q3)	75 55.0 (44.0, 64.0)	116 58.0 (50.0, 66.0)	191	0.109
**Sex, n (%)** Female Male	40 (32.0%) 35 (53.5%)	85 (68.0%) 31 (47.0%)	125 66	0.005
**Ethnicity, n (%)** Hispanic or Latino Not Hispanic or Latino Unknown/not reported	5 (62.5%) 67 (38.3%) 3 (37.5%)	3 (375%) 108 (61.7%) 5 (62.5%)	8 175 8	0.388
**Race, n (%)** White Black or African American Other	68 (39.5%) 4 (28.6%) 3 (60.0%)	104 (60.5%) 10 (71.4%) 2 (40.0%)	172 14 5	0.454
**ECOG PS, n (%)** 0 1 2 Unknown	47 (41.6%) 27 (37.5%) 0 (0.0%) 1 (50.0%)	66 (58.4%) 45 (62.5%) 4 (100.0%) 1 (50.0%)	113 72 4 2	0.230
**Cancer Site, n (%)** Breast/cervical/ovarian/uterine GI Other	12 (24.5%) 56 (44.1%) 7 (46.7%)	37 (75.5%) 71 (55.9%) 8 (53.3%)	49 127 15	0.048
**Neurotoxic agent, n (%)** Paclitaxel Oxaliplatin	15 (24.6%) 60 (46.2%)	46 (75.4%) 70 (53.8%)	61 130	0.004
**Years since enrollment (from January 2026)** N Median (Q1, Q3)	75 14.0 (13.0, 15.0)	116 15.0 (14.0, 15.0)	191	0.099
**Insurance payment method**, **n (%)** Medicaid or Medicare/Medicaid Medicare or Medicare/private insurance No means of payment (no insurance) Other Private insurance Self-pay (no insurance) Unknown	3 (42.9%) 11 (29.7%) 2 (40.0%) 1 (100%) 46 (37.4%) 6 (85.7%) 6 (54.5%)	4 (57.1%) 26 (70.3%) 3 (60.0%) 0 (0.0%) 77 (62.6%) 1 (14.3%) 5(45.5%)	7 37 5 1 123 7 11	0.090
**Length of trial, years [last enrolled—first]** N Median (Q1, Q3)	75 1.7 (1.7, 1.8)	116 1.7 (1.7, 1.8)	191	0.845
**County-level characteristics, N, median (Q1, Q3)** Percent in poverty 2023 (0–100%) Unemployment rate in 2013 (0–100%) Rural-Urban Continuum Code 2013 (1, 2, …9)	71 11.3 (9.0, 13.6) 71 6.8 (5.5, 8.5) 71 2.0 (1.0, 3.0)	111 12.1 (9.6, 15.2) 111 6.9 (5.5, 8.4) 111 2.0 (1.0, 3.0)	182 182 182	0.084 0.955 0.611
**Randomization ratio, n (%)** 2:1 (favoring experimental arm) 1:1	49 (45.8%) 26 (31.0%)	58 (54.2%) 58 (69.0%)	107 84	0.037
**Size of trial** ≥60 patients (N08CA, N08CB, A221805) <60 patients	61 (39.1%) 14 (40.0%)	95 (60.9%) 21 (60.0%)	156 35	0.922

Note: *p* values are two-sided and based on Fisher’s exact test for categorical variables and the Wilcoxon rank-sum test for continuous features. Not reported or unknown were excluded from statistical tests.

## Data Availability

Restrictions apply to the availability of these data. Data were obtained from and with permission from the Alliance for Clinical Trials in Oncology, the Academic and Community Cancer Research United (ACCRU) collaborative network, the Mayo Clinic Comprehensive Cancer Center, and the MD Anderson Cancer Center. De-identified patient data may be requested from the Alliance for Clinical Trials in Oncology via datasharing@alliancenctn.org if data are not publicly available. A formal review process includes verifying the availability of data, conducting a review of any existing agreements that may have implications for the project, and ensuring that any transfer is in compliance with the IRB. The investigator will be required to sign a data release form prior to transfer.

## References

[1] Staff N.P., Grisold A., Grisold W., Windebank A.J. (2017). Chemotherapy-induced peripheral neuropathy: A current review. Ann. Neurol..

[2] Colvin L.A. (2019). Chemotherapy-induced peripheral neuropathy: Where are we now?. Pain.

[3] Smith E.M., Pang H., Cirrincione C., Fleishman S., Paskett E.D., Ahles T., Bressler L.R., Fadul C.E., Knox C., Le-Lindqwister N. (2013). Effect of duloxetine on pain, function, and quality of life among patients with chemotherapy-induced painful peripheral neuropathy: A randomized clinical trial. JAMA.

[4] Li Y., Lustberg M.B., Hu S. (2021). Emerging Pharmacological and Non-Pharmacological Therapeutics for Prevention and Treatment of Chemotherapy-Induced Peripheral Neuropathy. Cancers.

[5] Tao Z., Chen Z., Zeng X., Cui J., Quan M. (2025). An emerging aspect of cancer neuroscience: A literature review on chemotherapy-induced peripheral neuropathy. Cancer Lett..

[6] Loprinzi C.L., Lacchetti C., Bleeker J., Cavaletti G., Chauhan C., Hertz D.L., Kelley M.R., Lavino A., Lustberg M.B., Paice J.A. (2020). Prevention and Management of Chemotherapy-Induced Peripheral Neuropathy in Survivors of Adult Cancers: ASCO Guideline Update. J. Clin. Oncol..

[7] Gewandter J.S., Brell J., Cavaletti G., Dougherty P.M., Evans S., Howie L., McDermott M.P., O’Mara A., Smith A.G., Dastros-Pitei D. (2018). Trial designs for chemotherapy-induced peripheral neuropathy prevention: ACTTION recommendations. Neurology.

[8] Belzung C. (2014). Innovative drugs to treat depression: Did animal models fail to be predictive or did clinical trials fail to detect effects?. Neuropsychopharmacology.

[9] Wager T.D., Atlas L.Y. (2015). The neuroscience of placebo effects: Connecting context, learning and health. Nat. Rev. Neurosci..

[10] Kinon B.J., Potts A.J., Watson S.B. (2011). Placebo response in clinical trials with schizophrenia patients. Curr. Opin. Psychiatry.

[11] Rutherford B.R., Pott E., Tandler J.M., Wall M.M., Roose S.P., Lieberman J.A. (2014). Placebo response in antipsychotic clinical trials: A meta-analysis. JAMA Psychiatry.

[12] Hafliðadóttir S.H., Juhl C.B., Nielsen S.M., Henriksen M., Harris I.A., Bliddal H., Christensen R. (2021). Placebo response and effect in randomized clinical trials: Meta-research with focus on contextual effects. Trials.

[13] Boussageon R., Howick J., Baron R., Naudet F., Falissard B., Harika-Germaneau G., Wassouf I., Gueyffier F., Jaafari N., Blanchard C. (2022). How do they add up? The interaction between the placebo and treatment effect: A systematic review. Br. J. Clin. Pharmacol..

[14] Sandra D.A., Olson J.A., Langer E.J., Roy M. (2023). Presenting a sham treatment as personalised increases the placebo effect in a randomised controlled trial. eLife.

[15] Frisaldi E., Vollert J., Al Sultani H., Benedetti F., Shaibani A. (2024). Placebo and nocebo responses in painful diabetic neuropathy: Systematic review and meta-analysis. Pain.

[16] Lavoie Smith E.M., Lee M., Scott M.R., Liu H., Hillman S., Rieken T., Wills R., Diaz-Cobo J., Chow S., Finnes H.D. (2026). Alliance A221805: Duloxetine to Prevent Oxaliplatin-Induced Chemotherapy-Induced Peripheral Neuropathy: A Randomized, Double-Blind, Placebo-Controlled Phase II Study. JCO Oncol. Adv..

[17] Wang X.S., Shi Q., Bhadkamkar N.A., Cleeland C.S., Garcia-Gonzalez A., Aguilar J.R., Heijnen C., Eng C. (2019). Minocycline for Symptom Reduction During Oxaliplatin-Based Chemotherapy for Colorectal Cancer: A Phase II Randomized Clinical Trial. J. Pain Symptom Manag..

[18] Zimmerman C., Atherton P.J., Pachman D., Seisler D., Wagner-Johnston N., Dakhil S., Lafky J.M., Qin R., Grothey A., Loprinzi C.L. (2016). MC11C4: A pilot randomized, placebo-controlled, double-blind study of venlafaxine to prevent oxaliplatin-induced neuropathy. Support Care Cancer.

[19] Loprinzi C.L., Qin R., Dakhil S.R., Fehrenbacher L., Flynn K.A., Atherton P., Seisler D., Qamar R., Lewis G.C., Grothey A. (2014). Phase III randomized, placebo-controlled, double-blind study of intravenous calcium and magnesium to prevent oxaliplatin-induced sensory neurotoxicity (N08CB/Alliance). J. Clin. Oncol..

[20] Pachman D.R., Dockter T., Zekan P.J., Fruth B., Ruddy K.J., Ta L.E., Lafky J.M., Dentchev T., Le-Lindqwister N.A., Sikov W.M. (2017). A pilot study of minocycline for the prevention of paclitaxel-associated neuropathy: ACCRU study RU221408I. Support Care Cancer.

[21] Shinde S.S., Seisler D., Soori G., Atherton P.J., Pachman D.R., Lafky J., Ruddy K.J., Loprinzi C.L. (2016). Can pregabalin prevent paclitaxel-associated neuropathy?—An ACCRU pilot trial. Support Care Cancer.

[22] Leal A.D., Qin R., Atherton P.J., Haluska P., Behrens R.J., Tiber C.H., Watanaboonyakhet P., Weiss M., Adams P.T., Dockter T.J. (2014). North Central Cancer Treatment Group/Alliance trial N08CA-the use of glutathione for prevention of paclitaxel/carboplatin-induced peripheral neuropathy: A phase 3 randomized, double-blind, placebo-controlled study. Cancer.

[23] Postma T.J., Aaronson N.K., Heimans J.J., Muller M.J., Hildebrand J.G., Delattre J.Y., Hoang-Xuan K., Lantéri-Minet M., Grant R., Huddart R. (2005). The development of an EORTC quality of life questionnaire to assess chemotherapy-induced peripheral neuropathy: The QLQ-CIPN20. Eur. J. Cancer.

[24] Lavoie Smith E.M., Haupt R., Kelly J.P., Lee D., Kanzawa-Lee G., Knoerl R., Bridges C., Alberti P., Prasertsri N., Donohoe C. (2017). The Content Validity of a Chemotherapy-Induced Peripheral Neuropathy Patient-Reported Outcome Measure. Oncol. Nurses Forum.

[25] Smith E.M.L., Zanville N., Kanzawa-Lee G., Donohoe C., Bridges C., Loprinzi C., Le-Rademacher J., Yang J.J. (2019). Rasch model-based testing of the European Organization for Research and Treatment of Cancer (EORTC) Quality of Life Questionnaire-Chemotherapy-Induced Peripheral Neuropathy (QLQ-CIPN20) using Alliance for Clinical Trials in Oncology (Alliance) A151408 study data. Support Care Cancer.

[26] Smith E.M.L., Knoerl R., Yang J.J., Kanzawa-Lee G., Lee D., Bridges C.M. (2018). In Search of a Gold Standard Patient-Reported Outcome Measure for Use in Chemotherapy- Induced Peripheral Neuropathy Clinical Trials. Cancer Control.

[27] Khan S., Khan S. (2020). Meta-analysis on One Proportion. Meta-Analysis: Methods for Health and Experimental Studies.

[28] Dibao-Dina C., Caille A., Sautenet B., Chazelle E., Giraudeau B. (2014). Rationale for unequal randomization in clinical trials is rarely reported: A systematic review. J. Clin. Epidemiol..

[29] Cho Y., Scott M.R., Wilson C.M., Daniel M., Odii C.O., Wang H.L., Carlee J., Kreider J., Smith E.M.L. (2025). Nurse Liaison Support to Safeguard the Scientific Rigor of Multisite Clinical Trials: Qualitative Findings From Communications Between Clinical Research Professionals. Cancer Nurs..

[30] Haidich A.B. (2010). Meta-analysis in medical research. Hippokratia.

